# Fetal and Newborn Management of Cloacal Malformations

**DOI:** 10.3390/children9060888

**Published:** 2022-06-14

**Authors:** Shimon E. Jacobs, Laura Tiusaba, Tamador Al-Shamaileh, Elizaveta Bokova, Teresa L. Russell, Christina P. Ho, Briony K. Varda, Hans G. Pohl, Allison C. Mayhew, Veronica Gomez-Lobo, Christina Feng, Andrea T. Badillo, Marc A. Levitt

**Affiliations:** 1 Division of Colorectal and Pelvic Reconstruction, Children’s National Hospital, Washington, DC 20010, USA; sjacobs@childrensnational.org (S.E.J.); ltiusaba@childrensnational.org (L.T.); ebokova@childrensnational.org (E.B.); tlrussell@childrensnational.org (T.L.R.); cho3@childrensnational.org (C.P.H.); bvarda@childrensnational.org (B.K.V.); hpohl@childrensnational.org (H.G.P.); acmayhew@childrensnational.org (A.C.M.); vgomezlo@childrensnational.org (V.G.-L.); cfeng@childrensnational.org (C.F.); abadillo@childrensnational.org (A.T.B.); 2 Division of General Surgery, Faculty of Medicine, Mu’tah University, Karak Governance, Mu’tah 61710, Jordan; tamadorshamaileh@gmail.com; 3 Pediatric and Adolescent Gynecology Program, Eunice Kennedy Shriver National Institute of Child Health and Human Development, Bethesda, MD 20892, USA

**Keywords:** cloaca, anorectal malformation, imperforate anus, VACTERL, hydrocolpos, hydronephrosis, colostomy, vesicostomy, anorectoplasty

## Abstract

Cloaca is a rare, complex malformation encompassing the genitourinary and anorectal tract of the female in which these tracts fail to separate in utero, resulting in a single perineal orifice. Prenatal sonography detects a few cases with findings such as renal and urinary tract malformations, intraluminal calcifications, dilated bowel, ambiguous genitalia, a cystic pelvic mass, or identification of other associated anomalies prompting further imaging. Multi-disciplinary collaboration between neonatology, pediatric surgery, urology, and gynecology is paramount to achieving safe outcomes. Perinatal evaluation and management may include treatment of cardiopulmonary and renal anomalies, administration of prophylactic antibiotics, ensuring egress of urine and evaluation of hydronephrosis, drainage of a hydrocolpos, and creation of a colostomy for stool diversion. Additional imaging of the spinal cord and sacrum are obtained to plan possible neurosurgical intervention as well as prognostication of future bladder and bowel control. Endoscopic evaluation and cloacagram, followed by primary reconstruction, are performed by a multidisciplinary team outside of the neonatal period. Long-term multidisciplinary follow-up is essential given the increased rates of renal disease, neuropathic bladder, tethered cord syndrome, and stooling issues. Patients and families will also require support through the functional and psychosocial changes in puberty, adolescence, and young adulthood.

## 1. Introduction

A cloacal malformation is characterized by a single perineal orifice where the urologic, gynecologic, and gastrointestinal tracts meet. It represents one of the most complex congenital anomalies of the pelvic organs in females, with an incidence of 1 in 50,000 births [1,2,3]. The cause of cloacal malformations is thought to be multi-factorial, including from genetic and environmental etiologies. Up to 10% of patients have a syndromic abnormality associated with a chromosomal or genetic anomaly [4]. In 30–70% of non-syndromic patients with anorectal malformations (ARMs), a combination of congenital anomalies of multiple systems known as the VACTERL (V-Vertebral, A-Anorectal, C-Cardiac, TE-tracheoesophageal-, R-Renal, L-Limb) association occurs. The diagnosis of the VACTERL association requires that three or more of these conditions are present [4,5,6]. Patients found to have a cloacal malformation require an expeditious workup of all additional congenital anomalies because they may be associated with life-threatening or urgent conditions. Herein, the key aspects of the newborn management of patients with cloaca are discussed.

## 2. Prenatal Diagnosis

Prenatal imaging findings associated with cloaca may be subtle. An accurate diagnosis can be made but requires a high degree of suspicion by knowledgeable fetal radiologists working alongside surgeons experienced with cloaca. The benefit of prenatal diagnosis, when possible, is the opportunity for providers to counsel parents in advance of delivery. Thorough counseling includes education regarding the anatomic variations in cloacal malformations, a discussion of any concomitant anomalies, outlining the priorities of neonatal management, and refining delivery planning to include immediate transfer to a tertiary care center. Early diagnosis allows families time to meet with experts in cloacal reconstruction who can accurately detail the surgical reconstruction and long-term functional outcomes from the colorectal, urological, and gynecologic perspectives. Prenatal diagnosis may also minimize potential complications by allowing time to develop a unified multidisciplinary plan for neonatal management that prioritizes key interventions and avoids delays in care due to unnecessary evaluations [7].

The most common findings on prenatal ultrasound (US) in patients with cloaca are abdominal/pelvic cystic masses, hydronephrosis, hydroureteronephrosis, oligohydramnios, ascites, and distended or obstructed bowel (Table 1). Additional anomalies that may be found include a two-vessel cord, distended bladder, echogenic bowel, multicystic renal dysplasia, ambiguous genitalia, and a range of cardiac anomalies [8]. Intraluminal calcifications, caused by urine mixing with meconium, can occur when stool flows antegrade into the bladder via the rectovaginal and then vaginourethral fistula. Fluid-filled dilated distal bowel with intraluminal calcifications or calcified meconium in the urinary tract are specific findings demonstrating communication between the distal bowel and the urinary tract [9,10,11,12].

**Figure 1:**
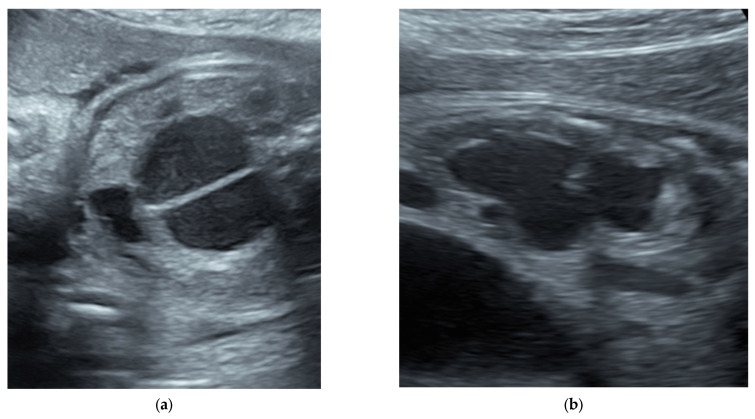
Fetal ultrasounds show: (**a**) hydrocolpos with longitudinal vaginal septum, and (**b**) hydronephrosis.

The differential diagnosis for a female fetus with an abdominal cystic mass should include ovarian cysts, enteric duplication cysts, abdominal cystic lymphangioma, intestinal atresia, megacystis microcolon intestinalis, and hydrocolpos due to imperforate hymen or urogenital sinus that leads to a distended vagina filled with fluid. The likelihood of a cloaca diagnosis in such a patient increases when two or three cystic structures can be identified—a bladder and a distended single vagina, a septate distended vagina only, without a visualized bladder, a bladder and distended septate vagina, or a distended distal rectum (Figure 1).

**Figure 2:**
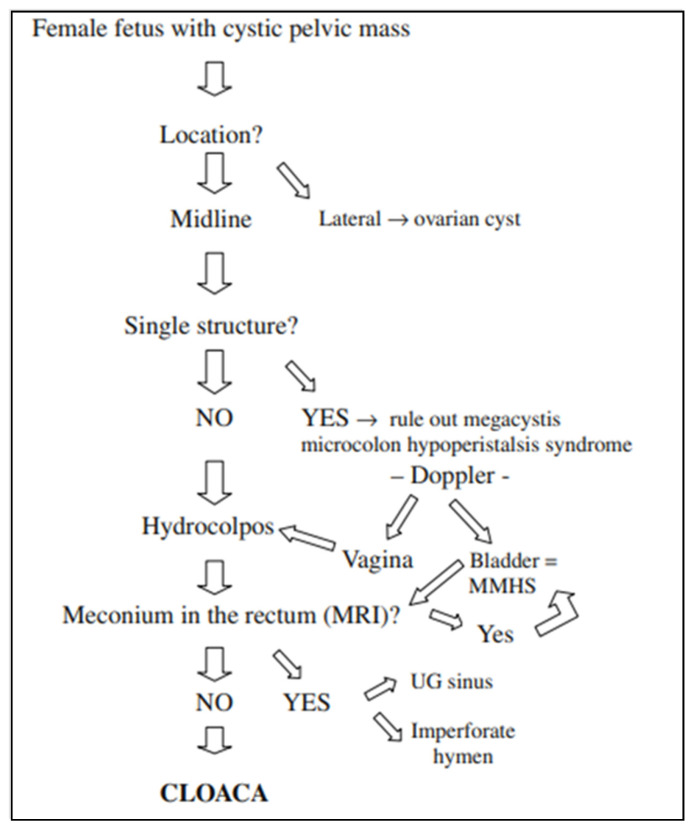
Algorithm for abdominal cystic mass in female fetuses. Adapted from Bischoff, et al. (2010) [11].

Ovarian cysts are the most common cystic lesions seen in female fetuses, but these are adnexal, lateral structures that occur with normal gastrointestinal and renal anatomy, and therefore can be distinguished from patients with cloaca. Bischoff et al. proposed the following imaging algorithm to work through the differential diagnosis of a cystic pelvic mass in a female fetus (Figure 2) [8]. A midline cystic structure may be the bladder, and with this finding one is obligated to rule out megacystis microcolon intestinal hypoperistalsis syndrome (MMHS). A color Doppler may visualize the paravesical vessels to help differentiate between MMHS and a hydrocolpos [13]. If the cystic mass is in fact the bladder, investigation for the presence of meconium in the rectum must be ascertained, which will be highly suggestive of MMHS. On the other hand, the absence of meconium in the rectum will support the diagnosis of a cloaca. A fetal MRI may be obtained to visualize this, as well as to scrutinize the remainder of the anatomy.

**Figure 3:**
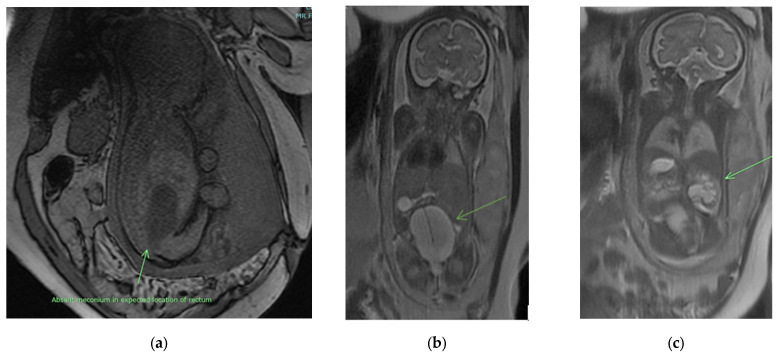
Prenatal MRI findings suggestive of a cloacal anomaly may include (as indicated by arrow): (**a**) absent meconium signal in the expected location of the rectum, (**b**) hydrocolpos with vaginal septum, and (**c**) hydronephrosis.

When US findings are suspicious for cloaca, a fetal MRI performed in the third trimester can delineate the anatomy of the fetus and confirm the diagnosis. MRI findings suggestive of cloaca include dilated distal bowel that does not extend below the bladder, lack of meconium signal at the expected rectal location, renal anomalies (most commonly hydronephrosis), abnormal appearance of the bladder, and hydrocolpos (Figure 3). An increased T2 signal in the distal bowel indicates the presence of fluid, while a decreased T2 or a bright T1 signal indicates meconium layering in the bladder. These findings are highly suggestive of a communication between the bowel and urinary systems [14].

The exstrophy–epispadias complex represents a more severe spectrum of congenital urologic, gynecologic, and gastrointestinal tract anomalies, which typically includes an abdominal wall defect with marked pubic diastasis. There is a high rate of associated spinal dysraphism, such as myelomeningocele. The exstrophy–epispadias complex can range from a mild defect of epispadias in 30% of patients, bladder exstrophy in 60%, or the most severe form of cloacal exstrophy in 10%, also known as the omphalocele–exstrophy–imperforate anus–spinal anomaly complex (OEIS) [15]. Cloacal exstrophy is estimated to occur in 1:200,000 to 400,000 births. Past survival rates were as low as 0% in 1900, 50% in 1960 when Rickham performed the first successful repair, 80–100% in the 1980s, and approached 100% by 2010 [16,17]. Prenatal diagnosis of cloacal exstrophy has evolved over time with improved differentiation between cloacal exstrophy and other anterior abdominal wall defects. The most common US findings are non-visualization of the bladder, a large midline infraumbilical anterior wall defect or cystic wall structure (persistent cloacal membrane), omphalocele or lumbosacral anomalies, and/or an “elephant trunk” representing prolapsed and intussuscepted ileum. Such findings should prompt further investigation of potential associated anomalies, such as lower extremity defects, renal anomalies, ascites, widened pubic arches, a narrow thorax, hydrocephalus, and a single umbilical artery [18,19]. Fetal MRI has specifically enhanced the ability to distinguish cloacal exstrophy from bladder exstrophy [20,21].

When a prenatal diagnosis of a cloacal malformation or cloacal exstrophy is made, caregiver counseling is a crucial intervention to assuage parental concerns. Devising a strategy for effective and tactful delivery of this difficult diagnosis is a key first step in counseling.

## 3. Counseling

The advantages of a prenatal diagnosis include the ability to discuss the viability of the fetus, offering the option of terminating the pregnancy, and establishing parental expectations of the prognosis for bowel, urinary, and gynecologic function [7]. In addition, the family is counseled regarding what to expect at delivery and in the initial postnatal period. In the case of a postnatal diagnosis of cloaca, counseling must occur synchronously with immediate evaluation and intervention, which may heighten the anxiety of the caregivers and healthcare providers. Transfer of the newborn, once stabilized, to a tertiary facility with experience in the management of these patients is recommended.

Although in-person counseling may be ideal, virtual visits allow families to receive expert counseling even when centers of excellence are not local, and web-based protocols have been described to help providers accomplish this [22,23,24,25]. Regardless of visit modality, the portion of the counseling session focusing on education should be comprehensive and include five key aspects:

### 3.1. Explanation of the Diagnosis

This should include a discussion of the suspected anatomy of the urethra, vagina, and colon connecting to a single perineal orifice, as well as the relative severity of cloaca compared to other ARMs in general. The provider should discuss the higher rate of associated anatomic anomalies and discuss patient-specific findings, such as VACTERL-associations, and the need for additional screening tests in the pre- and post-natal periods, and expectations for bowel and urinary control, and gynecologic function.

### 3.2. Monitoring during Pregnancy

Routine follow-up with the patient’s primary obstetrician, a maternal–fetal medicine specialist and prenatal sonography with the ability for advanced ultrasonographic monitoring continues through delivery particularly to monitor for oligohydramnios, renal status, and to assist with delivery planning. The frequency of ultrasound monitoring is dependent on the presence of coexisting fetal anomalies and/or oligohydramnios. The family can meet with the colorectal surgery, urology, gynecology, and neonatology teams for individualized counseling about perinatal interventions and long-term functional expectations.

### 3.3. Mode, Place, and Timing of Delivery

The diagnosis of cloaca alone does not necessitate a preterm delivery, preclude a vaginal delivery, or require delivery at an institution with advanced neonatal support. However, complicating factors such as coexisting fetal anomalies or severe oligohydramnios may warrant early and advanced delivery planning. Those with a high cardiopulmonary risk profile, for example, may be best suited for delivery at a hospital with level III or IV neonatal intensive care unit (NICU) capabilities. For others, the delivery may occur at the hospital of the family’s choice with a plan for expeditious transfer to a tertiary hospital with a NICU and subspecialty radiologic and surgical services. In those with anticipated spinal anomalies, particularly neural tube defects, or with severe abdominal distention, cesarean delivery may be recommended. However, for a majority of those with cloaca, vaginal delivery is safe and feasible [7]. Delivery planning is best made in conjunction with a maternal fetal medicine specialist.

### 3.4. Immediate Postnatal Care

After delivery, the priorities of care are to ensure respiratory and hemodynamic stability, including assessment for possible intubation. Additional examination and testing will be performed as part of a unified plan among surgical teams to triage the necessary interventions to achieve cardiopulmonary stability and secure egress of stool and urine, including drainage of a hydrocolpos, if present. Monitoring of renal function and management of azotemia may also be required. Until surgical interventions are completed, the newborn may remain nil per os (NPO) with parenteral nutrition for several days to weeks; therefore, necessitating a plan to store breastmilk if the mother plans to breastfeed.

### 3.5. Discharge and Initial Follow Up

Expected criteria required for discharge home are discussed, including appropriate recovery time following surgical interventions, a plan for oral or enteral feeds, regular stooling and colostomy care teaching, and adequate voiding or proficiency in intermittent catheterizations. Infants with cloaca requiring these maneuvers generally spend 2 to 3 weeks in the NICU, with a plan to perform reconstructive surgery in 3–12 months, depending on the infant’s growth and condition. The child will see their preferred pediatrician for routine well-visits to check growth and administer vaccines. Surgical subspecialty outpatient visits are scheduled to review interval testing such as sacral X-rays, serial renal ultrasounds, and spinal imaging. Plans for cloacagram and endoscopic evaluation for the delineation of the precise cloacal anatomy before reconstruction are outlined. Early patient-centered discussion of long-term functional outcomes helps to set expectations for families.

Routine communication between the caregivers and the high-risk obstetrician facilitates the implementation of the care plan at the delivery center and at the receiving center for the NICU transfer to manage the newborn.

## 4. Management in the Neonatal Period

Initial resuscitative measures of the neonate focus on achieving cardiopulmonary stability including a potential need for intubation. If a prenatal diagnosis of cloaca is not made, an imperforate anus may be discovered with a newborn exam, or even later in some cases. Vascular access is obtained to collect blood cultures, blood gas, hematologic and metabolic labs, and then to administer intravenous fluids as clinically indicated. Intravenous antibiotics such as ampicillin are recommended for prevention of urinary tract infections (UTIs) and broadened as required by clinical status. The team should prepare for a nasogastric sump tube insertion to evaluate for an esophageal atresia/tracheoesophageal fistula, present in 7–11% of patients with ARM [6,26]. Heightened vigilance to identify other VACTERL-associated anomalies during the physical exam is needed.

An echocardiogram is obtained to assess for cardiac defects, present in up to 40% of patients with ARM, and to determine if hemodynamic consequence may require intervention or additional considerations for general anesthesia administration for operative intervention [6,27,28].

**Figure 4:**
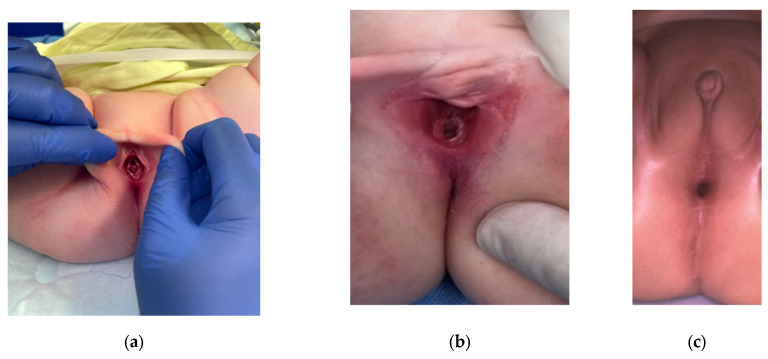
Female perineal exam shows: (**a**) proper technique to obtain maximal exposure (**b**), a single perineal opening at the expected urethral orifice location in a typical cloaca exam, and (**c**) a single perineal opening close to the expected anal orifice site, posterior to the urethral orifice and vestibule, consistent with a posterior cloaca.

Proper examination of a newborn perineum is often difficult to perform in a NICU, and the importance of good lighting with proper positioning cannot be overemphasized. Surgical loupes to closely examine the external anatomy can be useful, if available, especially for a premature newborn. The proper technique to perform a female perineal examination is to elevate the edges of the labia upward and outward towards the examiner’s shoulders to aid in the exposure of small, hidden orifices (Figure 4). A clitoris may appear hypertrophied as the clitoral hood and labia surround the single orifice, and this may be mislabeled as ambiguous genitalia; however, karyotyping will show XX and US will typically confirm Mullerian structures and the presence of bilateral ovaries [29]. A urogenital sinus with a normal anus, hence two orifices, is concerning for congenital adrenal hyperplasia with or without virilization, warranting electrolyte screening, endocrine workup, and karyotyping, whereas a single perineal orifice suggesting cloaca does not require endocrine workup. Another pitfall is to label the anorectal malformation as a rectovaginal fistula, which leads to rectal mobilization only at the time of surgical reconstruction, leaving the urogenital sinus untouched, requiring a complete revision in the future. A true rectovaginal fistula is exceedingly rare, and if a fistula is not seen in the vestibule in a female patient with an imperforate anus, a cloaca should be suspected [30].

**Figure 5:**
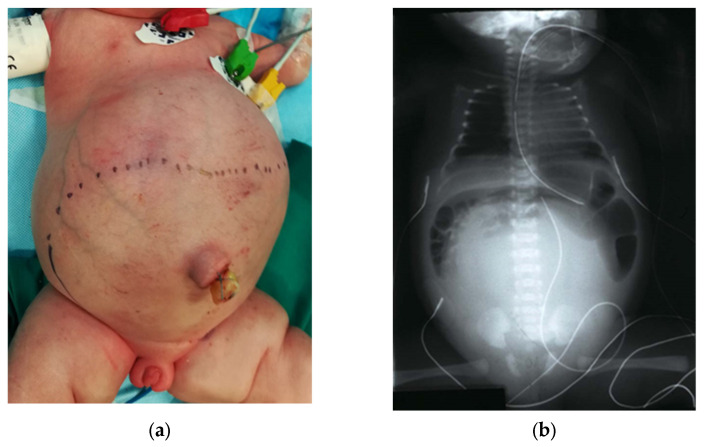
Hydrocolpos is found on: (**a**) a severely distended abdomen on physical examination at birth, and (**b**) a plain film radiograph showing a large opacifying structure with all bowel pushed superiorly, indicating a large pelvic mass.

A thorough physical exam also evaluates the abdomen for distention and the presence of a palpable lower midline mass, which may represent a hydrocolpos. This should be confirmed with bedside US, along with a check for concurrent hydroureteronephrosis [31]. An abdominal X-ray may show a pelvic mass opacifying a large portion of the abdomen (Figure 5). Up to a third of cloaca patients have hydrocolpos, which occurs when the vagina becomes distended by fluid, urine, or mucous [29,32]. The hydrocolpos can compress the posterior bladder and cause bilateral ureterovesical junction obstruction, which can result in life-threatening renal injury. Understanding that hydroureteronephrosis is likely caused by the hydrocolpos, the vagina must be drained before committing to any urological intervention such as nephrostomy, ureterostomy, or vesicostomy.

Initial attempts to drain the hydrocolpos are performed by passage of a catheter into the common channel, followed by US confirmation of vaginal decompression by the catheter placement. If two hemivaginas divided by a septum are present, which occurs in 25% of cloacal malformations, one must ensure that both vaginas are sufficiently drained [27]. If there is associated hydroureteronephrosis, renal labs and a renal/bladder US should be obtained 24–48 h after decompression to reassess the upper tract dilation and renal function. In rare cases, decompression will not resolve the upper tract dilation, which may warrant additional urologic intervention. A regular catheterization schedule may be needed if the patient does not void spontaneously via the common channel. Intermittent catheterizations may be continued until surgical repair if the caregivers can reliably perform them. If drainage via catheter is insufficient or not feasible, vaginostomy is typically effective in allowing for egress of urine and mucous. A tubed vaginostomy with a pigtail catheter can be placed under US guidance with an interventional radiologist, or a surgical tubed or tubeless vaginostomy may be fashioned at the time of colostomy creation [28,31]. Undrained or insufficiently drained hydrocolpos due to persistent or recurrent fluid accumulation poses a risk of persistent hydronephrosis as well as pyocolpos, in which scarring of the vagina could render it inadequate for future reconstruction, or in severe cases, it may perforate causing peritonitis [32].

When the hydrocolpos is resolved but the upper urinary tract dilation persists, a voiding cystourethrogram (VCUG) is helpful in diagnosing vesicoureteral reflux (VUR), ureteral ectopia, a long common channel, or a poorly emptying bladder [30]. It is usually difficult, so cystoscopy is needed [33]. If renal function is normal or only slightly impaired, the upper tract dilation may be monitored with repeat renal US prior to cloacal repair and afterward. In the case of renal dysfunction and/or azotemia secondary to ureteral obstruction, urinary diversion, such as by cutaneous ureterostomy, should be considered. A nuclear medicine functional renal scan, such as a MAG3 lasix renal scan, may be warranted to evaluate split renal function and discern for ureteral obstruction in the presence of persistent hydronephrosis or hydroureteronephrosis despite resolution of hydrocolpos. After 2 months of age, when the risk of kernicterus declines, amoxicillin may be switched to trimethoprim–sulfamethoxazole (2 mg/kg once daily), which provides better Gram-negative coverage. In very rare cases, a patient may have a long common channel, urethral atresia, or other bladder neck or distal urethral obstruction and the bladder does not drain. These patients require a vaginostomy, vesicostomy, or ureterostomy to drain the urine. A nephrostomy tube is almost never needed unless an obstruction at the level of the ureteropelvic junction is present.

Urologic anomalies are very common in patients with ARM. Those with cloacal malformations are especially susceptible, with urologic anomalies reported in more than 80% of cases [34]. Of these, VUR occurs in 50%, hydronephrosis in 30%, and solitary kidney in 15% [27]. Multicystic dysplastic kidney, ectopic ureter, duplex ureter, ureteropelvic junction obstruction, or primary obstructing megaureter may also be seen. Metabolic acidosis from poor renal function and/or urosepsis represents major sources of morbidity and mortality in newborns with ARMs. Furthermore, 50–75% of patients with cloaca have been reported to develop chronic kidney disease, with 17% developing end-stage renal disease [33,35]. Active involvement of a urologist in the care of newborns with cloaca is necessary for evaluation and management of urinary tract anomalies, prevention of UTIs, successful and consistent bladder drainage, and protection of renal function.

After initial stabilization of the patient’s cardiopulmonary status and drainage of a hydrocolpos (if present), the colostomy is created to divert the fecal stream. This can be performed at the same time as other indicated surgical procedures, such as tracheoesophageal fistula ligation or vaginostomy creation, typically in the first 24 to 48 h of life. A cystoscopy or vaginoscopy are not needed at the initial operation if the hydrocolpos and the urine are reliably draining. The ideal location in the colon for the stoma is the descending colon, just distal to the fixation to the left retroperitoneum and abdominal side wall, which helps to prevent prolapse, while leaving sufficient distal length for a future anorectoplasty [28]. In the vast majority of cases, the native vagina reaches the perineum. If it does not, vaginal replacement with small bowel or colon may need to be considered. For a colonic neovagina if adequate distal colonic length is available, the sigmoid loop can be used for a vaginal replacement, with a colo-colonic anastomosis and rectum brought down to the anoplasty, leaving the colostomy untouched to allow healing time. If the stoma is too distal, the proximal colostomy is mobilized and a short segment is divided and transposed to create the neovagina, and a more proximal colostomy is fashioned.

**Figure 6:**
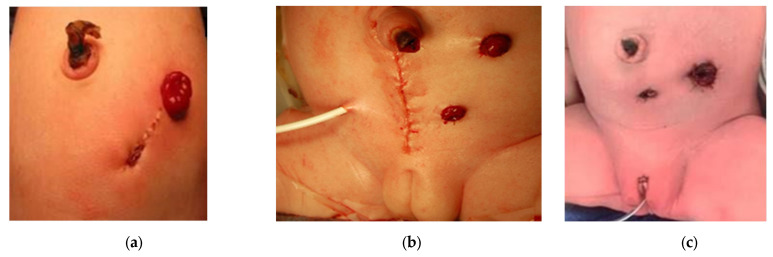
Panels show different techniques for colostomy creation in newborns with cloacal anomaly: (**a**) divided sigmoid colostomy with colostomy matured at the superior pole of the incision and a small, flat mucous fistula at the inferior pole with sufficient distance in between for pouching; (**b**) a low midline incision was made for surgical placement of a vaginostomy tube at the same time; therefore, separate incisions for the stoma ends were made like the laparoscopic technique shown in (**c**) a laparoscopic approach allows for two separate incisions.

If a divided colostomy is made, the mucous fistula is created small and flat to avoid prolapse, and sufficiently away from the colostomy with a skin bridge, to allow for ease of pouching of the proximal orifice to prevent skin breakdown and decrease spillover (Figure 6a) [35]. The marginal artery is often disrupted when the mesentery is divided, leaving the rectum supplied by inferior mesenteric artery branches, which occasionally need to be ligated in order to obtain sufficient length to reach the perineum. This leaves the bowel dependent on its intramural blood supply.

**Figure 7:**
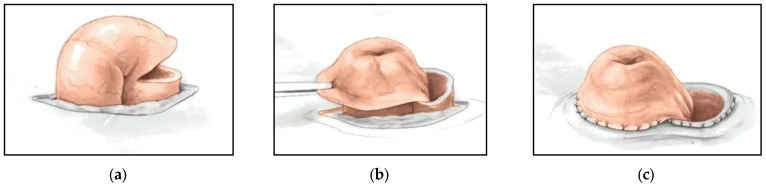
A Turnbull-style ostomy creation technique is demonstrated: (**a**) incision is made in the distal limb just above the skin; (**b**) Proximal limb is everted; (**c**) both limbs are sutured to the skin. Adapted from Beck et al. (2019) [39].

Loop colostomies preserve the blood supply and have the benefit of an easier reversal with shorter operative times [35,36]. In retrospective comparisons of loop to divided colostomies across ARMs, increased risk of prolapse has previously been reported with loop colostomy, particularly when placed in the transverse colon [27,36,37,38]. In these studies, while the numbers for cloaca are limited, the risk of UTI in an ARM patient, even in the presence of a rectourethral fistula, was not associated with the type of colostomy fashioned, and was driven by the underlying genitourinary anomaly. A Turnbull-style loop colostomy is a good option because it allows for minimal distal spillover, as the proximal limb is matured in a Brooke fashion, effaces the distal lumen, and preserves the marginal artery (Figure 7).

A low midline laparotomy may be performed to surgically drain a hydrocolpos at the same time as colostomy creation (Figure 6b). A tubed or tubeless vaginostomy, depending on its ability to reach the anterior abdominal wall, and the colostomy can be fashioned via separate incisions, similar to a laparoscopic approach [30]. Laparoscopy provides good visualization of the bowel orientation and position relative to its fixation points and allows for separate incisions for the proximal and distal ends (Figure 6c). The Mullerian structures can also be examined during laparoscopy. Routine oophoropexy is not performed as this creates the risk of limiting their blood supply without significant benefit.

**Figure 8:**
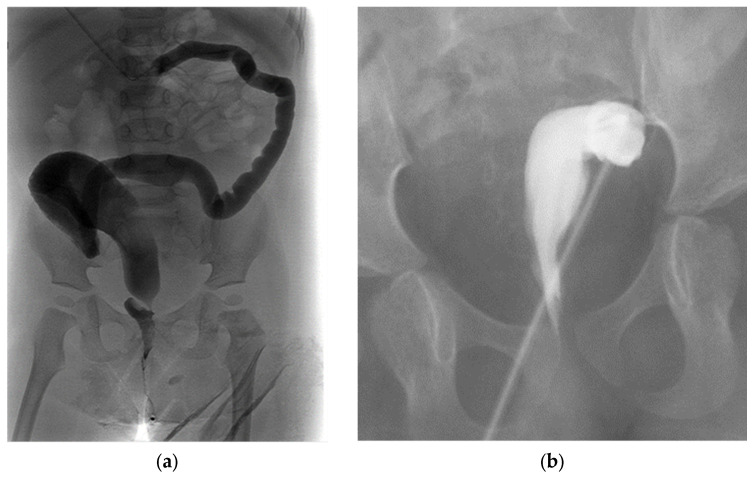
Distal colostogram images showing complications related to stoma and fistula placement, demonstrating (**a**) very long distal bowel from a transverse colostomy, and (**b**) very short segment of distal rectum from a colostomy placed too distally in the sigmoid colon.

A transverse colostomy can be associated with increased urinary absorption via the fistula, leading to acidosis, increased prolapse rates, and difficulty distending the distal colon and rectum on contrast imaging (Figure 8a) [31]. Placing a stoma too distally in the sigmoid colon can interfere with the future anorectoplasty, leaving inadequate bowel length between the stoma and anus, requiring a colostomy takedown and more proximal diversion (Figure 8b) [28].

After a colostomy is created, hydrocolpos is addressed (if present), and a bladder management strategy achieved, recovery can be rapid. Prolonged hospitalization often depends on comorbidities of the heart, lungs, or prematurity.

## 5. Post-Operative Course and Follow-Up

After the colostomy is functioning and the patient is clinically doing well, the patient may be fed. A newborn with a cloaca and a colostomy who is not doing well postoperatively usually has an undrained, obstructed urinary tract, with or without hydrocolpos. Therefore, the first study to perform in such patients should be an US, which can evaluate for hydronephrosis, megaureter, a distended bladder, or hydrocolpos. In the case of a loop colostomy or if both stomas are being pouched together, distal stool spillage may lead to a UTI [30]. Additional urologic studies, such as VCUG or MAG3 renal scan, may be performed and a plan for continued prophylactic antibiotics made. The caregivers need to be educated on stoma care, intermittent catheterizations of the common channel, flushing a vaginostomy tube to ensure patency, or care of a vaginostomy site, as applicable.

Any remaining components of a VACTERL work-up are completed before discharge, including a spinal US to assess for tethered cord, and plain radiographs looking for vertebral, radial, or other limb abnormalities. Particularly in a patient who struggles to drain their urinary tract, a neurogenic bladder arising from a spinal cord anomaly should be suspected. An inconclusive or definitively abnormal spinal US warrants a consultation with neurosurgery, who will assist with timing the repeat spinal US or MRI. Impaired bladder dynamics may be associated with poor development of the sacrum and severe caudal regression; therefore, dedicated sacral X-rays are performed after 3 months of age, after the sacral bones have ossified [40]. The final component that helps guide the conversation regarding prognosis for future bowel and bladder control is the severity of the anorectal malformation: a common channel shorter than 3 cm (moderate complexity) is associated with a better potential for volitional bladder emptying than a longer common channel (severe complexity). In the case of total urogenital mobilization of a moderate complexity cloaca, urethral length of greater than 1.5–2 cm is associated with a lower chance of urinary incontinence. Just prior to primary cloaca repair, the patient’s definitive anatomy and key measurements are elucidated during a multi-disciplinary evaluation including an exam under anesthesia, cystoscopy, vaginoscopy, and a contrast cloacagram [31,41,42,43].

If the Mullerian structures are not inspected during colostomy or vaginostomy creation, they may be evaluated during definitive repair, or if any future abdominal surgery is needed (such as colostomy closure or laparoscopy for a Malone appendicostomy). Greater than 50% of cloaca patients have Mullerian anomalies, and require longitudinal follow-up with a gynecologist [30,44,45]. Forty percent of patients develop obstructive menses; therefore, gynecologic consultation and sonographic evaluation around puberty and thelarche are essential [44,45]. Establishing a relationship early with a reconstructive gynecologist with experience in patients with cloaca is optimal.

The patient’s follow-up plan with the primary pediatrician, multiple surgical subspecialists, and outpatient testing are arranged, with precautions to seek urgent medical attention for issues such as fever, feeding intolerance, or decreased stoma output with abdominal distention, respiratory difficulties, lethargy, or purulent drainage with erythema of the surgical sites.

## 6. Conclusions

Prenatal diagnosis of a cloaca gives providers an opportunity to counsel parents about the likely diagnosis of cloaca, provide reassurance, and set expectations for the perinatal period and long-term functional outcomes. The first day of life focuses on the identification of associated anomalies and the triage of interventions to stabilize the infant’s cardiopulmonary status, divert the stool, secure drainage of hydrocolpos, and manage renal azotemia if present. Concomitant urologic anomalies should be anticipated, along with measures taken to diagnose and manage them in order to prevent renal damage and UTIs. A thoughtful colostomy with a plan in mind for future reconstruction, preserving the blood supply, and minimizing the risk of prolapse or distal spillage is essential. A collaborative model for planning and completing the reconstruction after the perinatal period is ideal to ensure the best outcomes for long-term bowel, bladder, and gynecologic function in these complex patients.

## Figures and Tables

**Table 1:** Rates of common anomalies identified by prenatal US in patients with cloaca ^†^.

Abdominal/pelvic cystic mass	52%
Hydronephrosis	49%
Oligohydramnios	26%
Ascites	22%
Distended bowel/bowel obstruction	18%

^†^ Adapted from Bischoff, et al. (2010) [8].

## Data Availability

Not applicable.
